# Establishing musculoskeletal oncology service in resource constrained country: challenges and solutions

**DOI:** 10.1097/IJ9.0000000000000050

**Published:** 2017-11-15

**Authors:** Obada Hasan, Akbar Zubairi, Zohaib Nawaz, Masood Umer

**Affiliations:** a The Aga Khan University Hospital (AKUH), Karachi; b The Combined Military Hospital Rawalpindi, Rawalpindi, Pakistan (CMH)

**Keywords:** musculoskeltal, tumors, oncology, developing country

## Abstract

The burden of orthopedic tumor surgery in Pakistan is not known. Similarly the number of procedures being performed for bone and soft tissue surgery are not known. This is even becoming more challenging where the existence of rules and regulations in health care are next to minimal. Furthermore data recording in our country and case registries hardly exist. Despite the lack of information and resources, with high disease burden on community, various providers provide surgical interventions every day in our settings. A lot of tumor surgery is still being done by general surgeons and general orthopedic surgeons who have little knowledge and update about musculoskeletal oncology principles. Lack of subspecialized centers and the high cost of such centers force the patients to visit these surgeons for a highly sophisticated problem like a bone tumor which is the disease of young bones. In this article we will emphasize on the difficulty in establishing an orthopedic tumor service in our part of the world and the consequences including delay in diagnosis, faulty course of management and later decline in functionality, disease progression and increased mortality. We will highlight the principles and stepwise approach of orthopedic tumor surgery and explain the difficulty encountered if these principles are not followed.

Malignant tumors are an overwhelming challenge to the orthopedic surgeons in a developing country due to inadequate awareness and limited diagnostic and therapeutic facilities. Another issue is the lack of the specialized orthopedic surgeons in tumor surgery in our side of the world, along with a scarcity of relevant published literature.

Orthopedic oncology is a highly sophisticated subspecialized field that requires a long and strenuous fellowship training in diagnosing and managing primary benign and malignant bone and soft tissue tumors. Even tumor surgery fellowships in North America typically produce no more than 12–15 new tumor surgeons each year^1^–^3^. Although general orthopedic surgeons maybe qualified to undertake surgical intervention of these tumors, but it is advisable to involve an orthopedic oncologist from the start to minimize the chances of bad outcome.

As primary bone sarcomas usually affect young individuals, an amputation could mean a lifelong dependency and disability hence decreased quality of life. In a society like ours where young people are responsible for entire households, it is imperative to restore their maximum physical capability and avoid amputation. This is now possible with a combination of recent innovations in understanding tumor biology and limb-salvage techniques supplemented by improved techniques in histopathology, radiology, radiotherapy, and medical oncology.

Because of lack of awareness on part of both patients and health care providers, interventions in tumor surgery are provided by many general orthopedic surgeons every day. This includes biopsy and tumor removal followed by referral to oncologist for chemotherapy and radiation. Coming from the developing country settings, knowing the prevalence and the scope of surgical disease is critical to planning further options. This stems largely from lack of awareness and financial gains to be credited after every procedure.

Impact of untreated or wrongly treated tumors, on disability, premature morbidity and mortality, presents a great challenge. Owing to an overall lack of knowledge and experience in this highly specialized field, surgeons end up performing surgeries with misplaced and miscalculated incisions, avoidable amputations or just treating patients on the basis of Tru-cut biopsy results from nonaccredited histopathology laboratory and untimely referrals to oncologists. A part of this problem lies with the patients who delay a visit to the surgeon or simply ignore the advice of referral to a specialist.

In efforts to avoid the above, development of treatment strategies have occurred in the past several years. Management starts stepwise from the time patient present in clinic, relevant imaging, staging, planned biopsy and incision site and size followed by appropriate treatment options. Interruption in this stepwise approach would lead to increased sufferings, disability, cost involved, and additional burden on the health care systems.

The purpose of this article is to review the principles of management of bone and soft tissue tumors, increase the awareness to this highly subspecialized field and emphasize on the need to develop such service in our part of the world despite the constraints.

## Discussion

Bone and soft tissue sarcomas are rare mesenchymal malignancies that arise in 2–4 per 100,000 head of population^4^. Overall survival following treatment of primary sarcoma now approaches 75% at 5 years, and surgery remains the mainstay of treatment^5^,^6^.

Historically, mainstay of managing these tumors was amputation. But as bone tumors are the disease of young bones, amputation increases the disability for those patients who have survival long enough to justify complex surgery. At our side of the world, society follows a joint family system where the entire family depends on one breadwinner. So when the latter is disabled, the entire family is paralyzed, both socially and economically. Thus it is imperative to restore their maximum physical capability and avoid amputation.

Surgery to resect the tumor followed by reconstructions to preserve function, mobility, and esthetics (limb-sparing surgery) has now replaced amputation as the primary form of surgical intervention^7^–^9^.

The major therapeutic goals are long-term survival, avoidance of local recurrence, maximizing function, and minimizing morbidity. Landmark trials conducted in the 1970s and 1980s at the National Cancer Institute showed equivalent survival outcomes between limb amputation and limb-sparing surgery combined with radiotherapy^10^,^11^.

Good outcome in limb saving procedures depends on multiple factors which was extensively studied and proved in literature like tumor size, depth, histologic grade, anatomic site, and margin size^12^,^13^. Older age has been reported to be associated with lower survival rates^14^,^15^. Older patients tend to present with larger and higher grade tumor which possibly result in increased local recurrences^16^.

At the other side, there are bad prognostic factors, yet avoidable. Examples include low degree of suspicion in plain radiographs, particularly if associated with history of trauma, or if suspected lesion is seen, doing the biopsy by nonexperienced personnel in the field of musculoskeletal tumors, late referral to the orthopedic oncologist and cancer center, and decreased awareness and knowledge by the patients, their relatives and even the physicians in account for the rarity of this disease.

**Figure 1:**
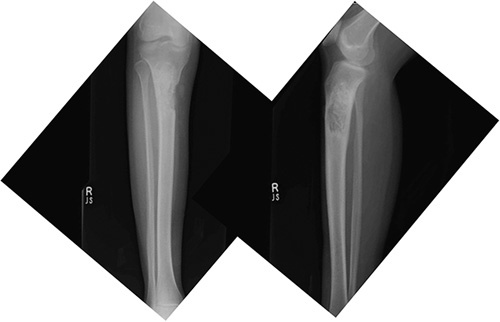
A 19-year-old girls presented with pain in right knee after a blunt trauma. Underwent aspiration and cytology showed no malignant cells. Eventually misdiagnosed as GCT. X-ray tibia anteroposterior and lateral views showing a lytic lesion at proximal tibia in which curettage and bone grafting was done. Final pathologic report is osteogenic sarcoma. Presented to our institute with recurrence/residual tumor. GCT indicates giant cell tumor.

Definite diagnosis is mandatory before any attempt at surgical intervention. In reality, this sometimes is difficult and patients with malignant bone disease can be misdiagnosed as having benign lesions. Tumors of an osteolytic behavior in their early stages can simulate giant cell tumor (**Fig. 1**). We recommend an open biopsy or frozen section diagnosis peroperatively. Such cases they present challenge even to the experienced orthopedic oncologist due to the violation of the tumor and the delay between the initial intralesional procedure and the proper definitive operation.

Another category of misdiagnosis reported is the radiographic findings misinterpreted as bone infection. They do not often go for biopsy and when healing is delayed it is attributed to the natural course of the disease^17^.

**Figure 2:**
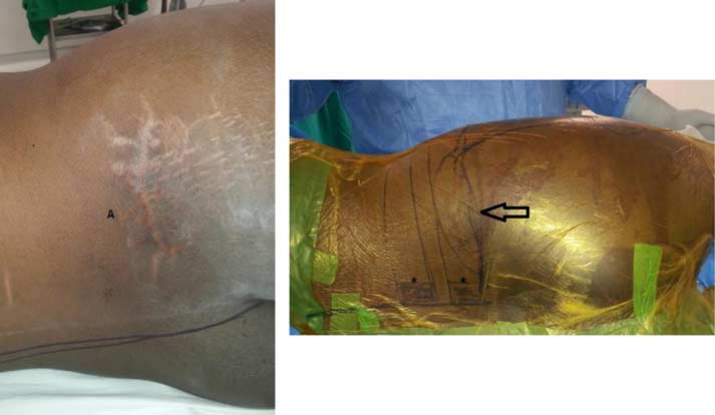
A 14-year-old boy with osteosarcoma over right buttock region, biopsied with large transverse incision. Clinical photograph with the patient in left lateral position, showing a large transverse incision (14 cm) just above the right buttock region done for an incisional biopsy for a suspected sarcoma (A). Right photograph showing the large incision (arrow) we were forced to do to include the previous large scar as well, which compromised the blood supply to the skin flap leading to marginal necrosis and second debridement and excision of necrotic skin. Asterisks showing L4 and L5 vertebrae levels.

A biopsy should be planned as carefully as the definitive procedure and should be done only after clinical and radiographic examinations are done. In principle, the same group that will be undertaking definitive treatment should perform the biopsy. Avoid transverse incisions because they increase the challenge for the limb salvage surgeon (**Fig. 2**).

It is worthy to mention here the principles of orthopedic tumors management and biopsy. A biopsy should be planned as carefully as the definitive procedure and should be done only after clinical and radiographic examinations are done. Biopsy incision and tract is considered contaminated with tumor cells and should be in the excised specimen. Transverse incisions should be avoided because they are extremely difficult or impossible to excise with the specimen. If a drain is used, it should exit in line with the incision so that the drain track also can be easily excised en bloc with the tumor^18^.

Our limited knowledge on this issue arises from the epidemiological factors that included neglect, unawareness of the problem, low socio economic status and financial burden involved in seeking treatment, and limited diagnostic facilities. On top of that is the lack of subspecialized centers and orthopedic tumor surgeons. This leads to increasing disability and impairing the quality of life of our patients. By and large, such tumors carry poor prognosis with high morbidity and mortality.

Cause of delay in seeking medical advice was neglect by the patient and family due to financial constraints, culture, lack of access to health care facilities, consultation with traditional bone settlers and even misdiagnosis by general orthopedic surgeons.

Giving the rarity of these tumors, along with their wide range of occurrence in any part of the body, they are resected by physicians other than orthopedic oncologists with nonstandardized techniques and without keeping a suspicious of malignancy in mind and safe margins^19^–^21^.

Proper management of these tumors require a multidisciplinary approach involving a qualified orthopedic oncology surgeon who is familiar with limb-salvage procedures supplemented by improved techniques in histopathology, radiology, radiotherapy, and medical oncology. Early referral of these patients plays a vital rule for better outcome. Because this is such an uncommon disease, it is so helpful to have guidelines like the National Comprehensive Cancer Network (NCCN) guidelines, because you are likely not going to see so many patients within your practice. Guideline states that when a patient younger than 40 years old presents to you with a bone pain, and you see an abnormal suspicious lesion on plain radiographs you should refer them to an orthopedic oncologist for biopsy and biopsy should be performed at the treating institution. If the patient is elder than 40 years, then they should be worked up for potential bone metastasis^22^. Davis and colleagues compared the outcomes of patients treated primarily in a cancer center versus those treated at noncancer centers who were referred after an unplanned excision. They found that the rate of a local recurrence was higher in the unplanned surgical excision group, particularly patients with a residual tumor in the reresected specimen^23^. Goodlad and colleagues reported 95 reresections in patients initially treated in noncancer centers from a series of 236 patients with soft tissue sarcomas. They found that 59% of those patients who had undergone unplanned resections had inadequate margins after the reresection^24^.

At our institute, 135 operated patients with soft tissue sarcomas evaluated for outcomes, in terms of local recurrence and metastasis rate, of reexcision following unplanned excision of the tumor at prereferral hospital, results compared with those of first-time planned surgery. We reported that local recurrence, metastasis, and mortality rates were higher in patients who underwent unplanned resections (21.4% vs. 14.3%, 13.7% vs. 8.3%, 13.7% vs. 9.5%, respectively)^18^. Lewis et al^25^ showed that disease specific metastasis-free survival rate was lower in patients who underwent reresection as compared with those who underwent planned primary surgery.

The clinicians and the pathologists handling management responsibility must have high index of suspicion as to the nature of bone lesion in order to establish the diagnosis of bone tumors. This applies specifically to orthopedic tumors because behavior of such tumors is less aggressive than other visceral tumors in body so the aim of the tumor surgeon is usually toward limb salvage and better quality of life.

Limb-sparing surgery is the technique of choice for surgical management of limb sarcomas. In comparison to amputation, limb-sparing surgery has the same overall survival rate, higher patient satisfaction, lower energy expenditure for walking and a lower cost to the community^26^. Innovative techniques are available that may result in a functional limb^27^,^28^.

Reconstruction techniques at our side of the world are technically demanding and very expensive for our patients who pay the whole medical course and services out of their own pocket. We reviewed 40 consecutive pediatric patients, aged 16 years or younger, with locally aggressive or malignant bone tumors treated with tumor resection, autoclaving and reimplantation of the orthotropic auto graft. Vascularized or nonvascularized fibular graft was used as a biological adjunct and fixation done with plate. We recommended a low cost alternate using the patient’s own autoclaved tumor bone for skeletal reconstruction. It consists of excision, sterilization, and reimplantation. Having the advantages of biological reconstruction with a “custom fit” segment, providing anatomic site for muscles and tendons reattachment, avoiding immunological response or transmission risks, no bone banking required, cheap, convenient for the surgeon, less operating time comparing to other reconstructive procedures and having higher incidence of integration and healing than allografts. We have used it successfully in both pediatric and adult populations^29^,^30^.

Bone and tissue bank forms a very essential back-up service for any musculoskeletal oncology service. Unfortunately we do not have a custom of organ donation by the deceased; hence bone banking is virtually nonexistent. Secondly, using implants in pediatric tumor reconstruction is an extremely costly solution in our society. Growing implants may not provide all solutions to difficult pediatric problems. We have successfully used and published the use of fresh parental fibular allograft in reconstruction after limb salvage surgery. This innovative technique is again with low cost, and with minimal morbidity. We hardly saw any tissue reaction in any of our patients^31^.

Data management in the form of a formal tumor registry is also being practiced within our section of orthopedics. This has helped us put out numerous publications in the field of musculoskeletal oncology^32^–^47^. We feel that now this experience needs to be replicated on a national level and we are making efforts in this regard.

We emphasize that the experts who received advanced training in Orthopedics tumor surgery should deal such cases. Orthopedics societies should guide surgeons to refer such cases to the concerned to save the lives and limbs of our patients.

Development of expert manpower resources was the first challenge. One of our surgeons (senior author) went abroad on Faculty Development Award provided by the hospital. He went to Mayo Clinic, Rochester, MN and then to Rizzoli Institute, Bologna, Italy. This experience helped him learn the latest techniques of limb salvage surgery. Since then he has been in various musculoskeletal oncology centers around the world to keep pace with latest developments in this field. Similarly, our histopathologist, went to Cleveland Clinic and then Mayo Clinic to gain deeper insight into pathologic diagnosis of bone and soft tissue disorders. Likewise we have teamed up with a radiologist who specializes in musculoskeletal magnetic resonance imaging and he is our partner in orthopedic tumor board. These key manpower developments have helped us build a team of experts who collectively decide the course of action required for a particular sarcoma patient.

It was after a long effort that we were able to establish a musculoskeletal tumor board many years back. This paved the way for combined management of such challenging cases. There was no example existing in the whole country when we started this activity. Now at least we have 3 orthopedic tumor boards running in different institutions in different parts of our country. We now have a regular monthly scheduled multidisciplinary tumor board meeting separately for adult and pediatric patients. This board includes the orthopedic tumor surgeon, senior medical and radiotherapy oncologists, pathologist, radiologist, residents and medical students as well. This board provides insights and feedback and approved beneficial for the patients, and for all members. Above all we believe that it is the right of every patient to be presented in such highly professional meeting and we invite cases from other institutions all over the country to be discussed in this board.

## Conclusions

This review tried to highlight some of the causes of neglect in malignant bone and soft tissue tumors in our side of the world. We require a concrete effort from the Orthopedics societies and the government to create awareness among general physicians and surgeons to know the consequences of such neglect and early referral to the orthopedics tumor surgeons to save loss of extremities. Considering their very small numbers, we need to train more orthopedic oncology surgeons in Pakistan. They need to join hands in developing and maintaining a national Orthopedics tumor registry. This will help produce publications and reflect on our work periodically. Tumor board is very effective in increasing the knowledge and experience and above all improves patients’ outcome.

## Ethical approval

It is a review article, approved for exemption by ERC.

## Sources of funding

None.

## Author contribution

O.H.A.H.: initial draft, writing of final manuscript. A.Z.: editing. Z.N.: contribution of clinical pictures and editing. M.U.: supervision of all steps of manuscript writing.

## Conflict of interest disclosure

The authors declare that they have no financial conflict of interest with regard to the content of this report.

## Research registration unique identifying number (UIN)

Not applicable as this is a review article.

## Guarantor

Obada Hasan and Masood Umer.
